# Multicenter evaluation of fast multiplex PCR for detection of pathogens in lower respiratory tract infections

**DOI:** 10.3389/fcimb.2025.1643991

**Published:** 2025-10-02

**Authors:** Ling Wang, Jingyao Cai, Lizhong Dai, Weimin Miao, Ziyang Li, Wei Cao, Sisong Huang, Mei Sun, Lehuan Xia, Xixin Jiang, Pingbang Wang, Jianhua Pan, Qinghe Yang, Anqun Yang, Min Hu

**Affiliations:** ^1^ Department of Laboratory Medicine, The Second Xiangya Hospital of Central South University, Changsha, Hunan, China; ^2^ Sansure Biotech Inc., Changsha, Hunan, China; ^3^ Department of Laboratory Medicine, Chenzhou Third People’s Hospital, Chenzhou, Hunan, China; ^4^ Department of Laboratory Medicine, Yueyang People’s Hospital, Yueyang, Hunan, China; ^5^ Department of Laboratory Medicine, The People’s Hospital of Liuyang, Liuyang, Hunan, China; ^6^ Department of Laboratory Medicine, Changsha Central Hospital, Changsha, Hunan, China; ^7^ Department of Laboratory Medicine, The Second People’s Hospital of Hunan Province Huaihua City, Huaihua, Hunan, China

**Keywords:** multicenter evaluation, lower respiratory tract infections, pathogen detection, multiplex PCR, semi-quantitative result

## Abstract

**Background:**

This study aimed to compare the diagnostic performance between bacterial culture and a rapid multiplex PCR assay (Respiratory Pathogens Multiplex Nucleic Acid Diagnostic Kit) for detecting six bacterial (Pseudomonas aeruginosa, Klebsiella pneumoniae, Staphylococcus aureus, Streptococcus pneumoniae, Haemophilus influenzae, Legionella pneumophila) and six viral targets (Influenza A/B, Respiratory syncytial virus, Adenoviruses, Human rhinovirus, Mycoplasma pneumoniae) in 728 bronchoalveolar lavage (BAL) specimens.

**Methods:**

This multicentric observational study was conducted at six comprehensive large hospitals in Hunan Province, China (May to October 2023) and assessed performance of mPCR kit by comparison with conventional culture method.

**Results:**

The mPCR kit detected ≥1 pathogen in 628 specimens (positivity rate: 86.3%), demonstrating positive percentage agreement (PPA) of 84.6% (95% CI: 76.6-92.6%) and negative percentage agreement (NPA) of 96.5% (95% CI: 96.0-97.1%) versus culture. Notably, semi-quantitative concordance was 79.3% (283/357) for culture-positive specimens. Multiple pathogens were detected by mPCR in 144 samples (19.8%). Lower Ct values (≤30) correlated strongly with culture positivity.

**Conclusions:**

The mPCR kit is a simple and rapid molecular test that could complement conventional culture method for improvement of diagnosis accuracy of Lower respiratory tract infections.

## Introduction

Lower respiratory tract infections (LRTI) are among the leading global causes of morbidity and mortality globally, posing a significant health burden (Collaborators, 2018). Timely detection of the underlying pathogens and determination of their susceptibility profiles are paramount for effective treatment and improved clinical outcomes (Iregui et al., 2002). Current diagnostic routines, including culture-based methods for pathogen identification and antimicrobial susceptibility testing (AST), often take over 48-72 hours to yield results (Torres et al., 2016). Furthermore, these methods may fail to detect clinically significant pathogens due to prior antibiotic exposure or stringent growth requirements (Prats et al., 2002). Therefore, there is a need for molecular diagnostic testing that yields rapid and reliable results to guide effective treatment and prevention decisions.

The diagnosis of LRTI has greatly advanced in recent years, thanks to the development of numerous respiratory viral and bacterial panel assays that are now commercially available (Ramanan et al., 2018). A Respiratory Pathogens Multiplex Nucleic Acid Diagnostic Kit is a fully automated multiplex PCR assay for identifying several typical bacterial pathogens, respiratory viruses, directly from sputum, endotracheal aspirate (ETA), and bronchoalveolar lavage fluid (BALF) specimens in approximately one hour. The assay provides semi-quantitative results for 12 typical respiratory pathogens.

The aim of this large retrospective observational multicentric study was to evaluate analytical performances of the fast multiplex PCR assay for the detection of pathogens by comparison with findings obtained by conventional culture methods.

## Materials and methods

### Study subjects

This investigator-initiated, multicenter, retrospective observation study was conducted at six comprehensive large hospitals in Hunan Province, China.

This study was performed with 728 BALF specimens collected from May to October. The specimens were stored at -80 °C following testing using routine microbiological methods. This study has been approved by the research and ethics committee of the Second Xiangya Hospital of Central South University (No.2023100).

### Routine conventional culture

Bacterial culture was performed by inoculating bronchoalveolar lavage fluid (BALF) onto three selective and differential media: Blood agar–A nutrient-rich medium for broad-spectrum bacterial cultivation, particularly optimal for Gram-positive cocci and streptococci (including Staphylococcus aureus and Streptococcus pneumoniae identified in this study), enabling observation of pathogen diversity and growth trends;Chocolate agar–Enriched with NAD and hemin to support fastidious organisms, specifically targeting Haemophilus influenzae in our cohort;HE agar (Hektoen Enteric Agar)–A selective medium for Gram-negative bacilli isolation, critical for detecting Pseudomonas aeruginosa and Klebsiella pneumoniae through bile salt inhibition and sugar fermentation indicators. Plates were inoculated and streaked using calibrated loops in accordance with local protocols to achieve a semi-quantitative culture result. Inoculated media were incubated at 35°C in a 5% CO_2_ atmosphere and were examined daily for bacterial growth. Following incubation, a sterile, disposable inoculation loop was used to transfer sufficient colonies of a pure culture from those subculture media to a 96-spot polished steel target plate (Bruker Daltonics, Bremen, Germany) for MALDI-TOF MS analysis. This study defined RCM no bacterial growth as: after three days of incubation at 35°C in a 5% CO_2_ atmosphere, no colony growth was observed on any plates.

### Respiratory pathogens multiplex nucleic acid diagnostic kit

The Respiratory Pathogens Multiplex Nucleic Acid Diagnostic Kit simultaneously detects six bacterial targets (Pseudomonas aeruginosa, Klebsiella pneumoniae, Staphylococcus aureus, Streptococcus pneumoniae, Haemophilus influenzae, Legionella pneumophila), six viral targets (Influenza A virus, Influenza B virus, Respiratory syncytial virus, Adenoviruses, Human rhinovirus, Mycoplasma Pneumonia). The Respiratory Pathogens Multiplex Nucleic Acid Diagnostic Kit is a closed, pouch-based, syndrome-specific multiplex PCR test intended for use with the Hongshi SLAN-96P Fully automated medical PCR analysis system and Life Technologies QuantStudio™ 5 Fluorescence PCR machine. From approximately 1mL of specimen, it includes all steps and provides results in around 75 minutes. The panel allows the detection of six typical bacteria and six viruses. The positive value of the Ct was 39.

### Data analysis

Results from conventional culture method and mPCR were compared for detection of bacteria. For each microorganism identification, a result was considered as true positive (TP) or true negative (TN) if results of mPCR and routine conventional culture were concordant. In the first analysis, routine conventional culture was defined as the reference standard, meaning that a microorganism identified only by the mPCR and not by routine conventional culture was considered as a false positive (FP) and conversely a target found by conventional methods and not by mPCR was considered as a false negative (FN). Categorical variables were described as frequencies and percentages and compared using Chi-square test. *P*-values less than 0.05 were considered statistically significant. Statistical analyses were performed using SPSS software (version 27.0 for Windows; Chicago, Illinois, USA). Figures were made using GraphPad Prism (version 9.3.0).

## Results

### Study population and samples

**Figure 1:**
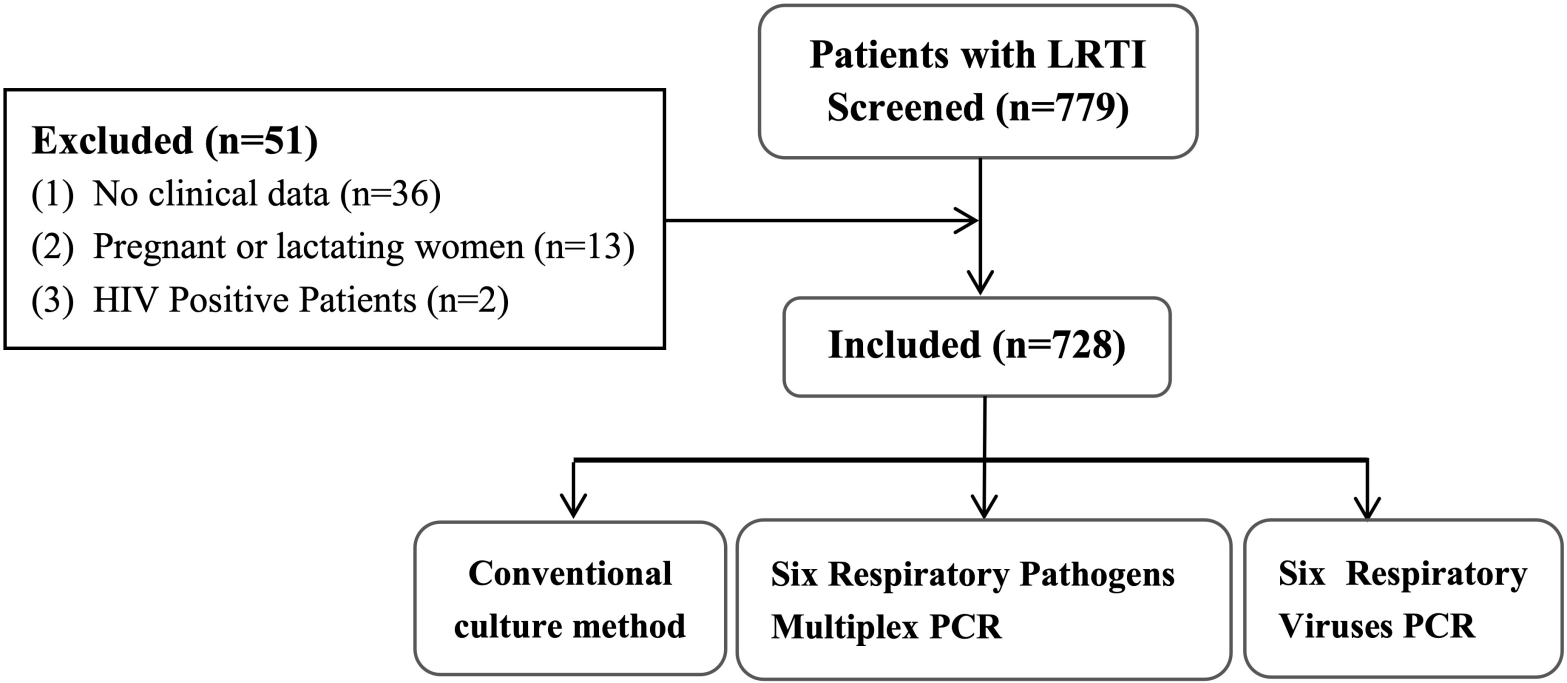
Flow chart of this study.

**Figure 2:**
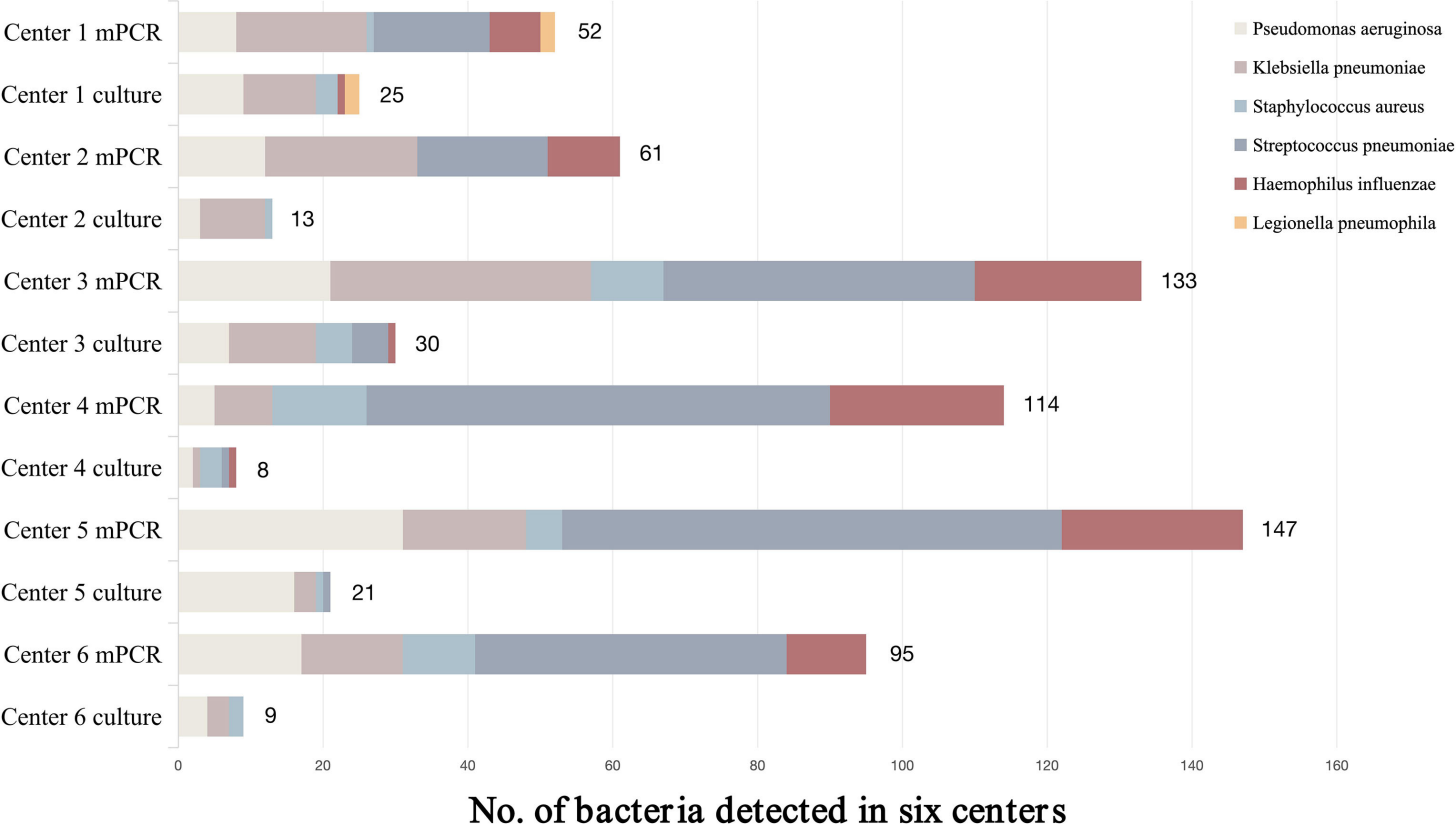
Number of bacteria in six centers.

A total of 779 BALF specimens were analyzed, of which 51 had to be excluded due to invalid results (**Figure 1**). The average patient age was 65.2 years, and 465 patients (63.9%) were male. Of the 728 with interpretable results, culture and mPCR yielded positive results in 103 (14.15%) and 405 (55.63%), respectively. **Table 1** demonstrates that the differences in pathogen detection rates between RCM and mPCR methods across all centers are statistically significant. In addition, **Figure 2** showed that the number of pathogens in different centers using mPCR and culture methods.

**Table 1:** Overview of testing of the 728 BALF specimens.

Centers	BALF specimens (n=728)	Bacterial targets (no. of positive samples)		
		RCM (n=728)	mPCR (n=728)	*P*-value
Center 1	137(18.8%)	24	39	0.01^*^
Center 2	100(13.7%)	13	42	0.007^*^
Center 3	145(19.9%)	28	86	0.01^*^
Center 4	123(16.9%)	8	79	0.001^*^
Center 5	125(17.2%)	21	95	0.004^*^
Center 6	100(13.7%)	9	64	0.002^*^
Total, n (%)	728	103(14.15%)	405(55.63%)	0.005^*^

Center 1: The Second Xiangya Hospital of Central South University, Changsha, Hunan, PR China; Center 2: Chenzhou Third People’s Hospital, Chenzhou, Hunan, PR China; Center 3: Yueyang People’s Hospital, Yueyang, Hunan, PR China; Center 4: The People’s Hospital of Liuyang, Liuyang, Hunan, PR China; Center 5: Changsha Central Hospital, Changsha, Hunan, PR China; Center 6: The Second People’s Hospital of Hunan Province Huaihua City, Huaihua, Hunan, PR China. *P-value< 0.05 were considered statistically significant.

**Table 2** presents the number of samples in which multiple pathogens were detected by the mPCR and conventional culture methods. Of the 728 samples included in the study, multiple pathogens were detected in 144 samples (19.8%) using the mPCR, ranging from two pathogens in 115 samples (15.8%) to four pathogens in 8 sample (1.1%). Conversely, the culture method detected two pathogens in four samples (0.5%).

**Table 2:** Number of samples in which multiple pathogens were detected using the respiratory pathogens multiplex nucleic acid diagnostic kit and culture methods.

Culture methods	Respiratory pathogens multiplex nucleic acid diagnostic kit					Total
	0	1	2	3	4	
0	318	209	83	12	5	627
1	8	49	31	7	2	97
2	0	0	1	2	1	4

0 = no pathogens detected, 1 = one pathogen detected, 2 = two pathogens detected.

### Findings of the Respiratory Pathogens Multiplex Nucleic Acid Diagnostic Kit

The frequencies of the detected bacteria are presented in **Supplementary Table S1**. 105 bacteria were isolated using culture methods, with Pseudomonas aeruginosa (5.63%) and Klebsiella pneumoniae (5.36%) isolated most frequently, followed by Streptococcus pneumoniae (0.96%) and Haemophilus influenzae (0.55%). It can be seen that the positive rate of pathogens in conventional culture method was lower.

Meanwhile, the Respiratory Pathogens Multiplex Nucleic Acid Diagnostic Kit detected 628 pathogens in **Supplementary Table S1** (according to the instructions of the reagent kit, the result is positive when Ct is less than 40). The kit detected Streptococcus pneumoniae (30.08%) most frequently, followed by Klebsiella pneumoniae (15.11%), Pseudomonas aeruginosa (13.19%) and Haemophilus influenzae (12.91%). It can be seen that the results between the mPCR method and conventional culture method are quite different.

Interestingly, we found if the Ct value is reduced, the result is positive when Ct is less than 30, and the positive rate of mPCR detection is basically consistent with conventional culture method. While **Table 3** demonstrates improved concordance between mPCR (Ct<30 threshold) and culture methods for bacterial pathogens, statistical analysis revealed significant differences (P<0.05) in detection rates across all targeted bacteria except Legionella pneumophila. Most notably: Streptococcus pneumoniae showed the largest discrepancy (*P* = 0.003). Haemophilus influenzae detection differed significantly (*P* = 0.01). **Table 3** showed that the kit detected 286 pathogens. The most frequently detected typical bacteria were Streptococcus pneumoniae (7.14%), Pseudomonas aeruginosa (6.6%) and Klebsiella pneumoniae (5.63%). Meanwhile, the multiplex PCR assay detected Mycoplasma pneumoniae as the predominant viral pathogen (63.8%, 44/69), with adenovirus (13.0%) and human rhinovirus (10.1%) as minor contributors among 69 viral identifications.

**Table 3:** Pathogens identified using the respiratory pathogens multiplex nucleic acid diagnostic kit (Ct<30) and culture-based methods.

Category	RCM+, n (%)	PCR+(Ct<30), n (%)	*P*-value
Bacterium			
Pseudomonas aeruginosa	41(39.0%)	48(7.6%)	0.5
Klebsiella pneumoniae	39(37.1%)	41(18.9%)	0.8
Staphylococcus aureus	14(13.3%)	37(5.9%)	0.07
Streptococcus pneumoniae	7(6.7%)	52(8.3%)	0.01^*^
Haemophilus influenzae	4(3.8%)	36(5.7%)	0.03^*^
Legionella pneumophila	0	3(0.5%)	/
Viruses			
Influenza A virus	/	2(2.9%)	/
Influenza B virus	/	2(2.9%)	/
Respiratory syncytial virus	/	5(7.2%)	/
Adenovirus	/	9(13.0%)	/
Human rhinovirus	/	7(10.1%)	/
Mycoplasma pneumoniae	/	44(63.8%)	/
Total	105	286	/

*P-value < 0.05 were considered statistically significant.

### Correlation between Ct values and bacterial count based on culture method

The mPCR Ct values and bacterial amounts based on culture method are shown in **Table 4**. This diagnostic kit detected bacterial targets of Ct<30 if the bacteria were positive by the culture method. Conversely, Ct values determined by this diagnostic kit ranged from ≥ 30 in culture-negative samples.

**Table 4:** Comparison between respiratory pathogens multiplex nucleic acid diagnostic kit results and bacterial count assessed by culture.

Culture amount (semi-quantitative)	The respiratory pathogens multiplex nucleic acid diagnostic kit				Concordant %
	Not detected	30≤ct<40	25≤ct<30	Ct<25	
No significant amount	177	88	27	8	265/300 88.33%
Significant amount	8	11	25	13	38/57 66.67%
Moderate ^a^	7	6	8	3	8/24 33.33%
Many ^b^	1	5	17	10	10/33 30.30%

a Growth up to the second quadrant of the plate.

b Growth up to the third or fourth quadrant of the plate.

Among 300 specimens that exhibited no significant bacterial growth in culture, 265 (88.3%) gave negative results using mPCR. Conversely, among 57 cultures that exhibited significant amounts in culture, 38 (66.67%) specimens showed Ct values less than 30 of bacterial nucleic acids using mPCR. In the cultured positive samples, the bacteria growth up to the second quadrant in 24 plates, with 8(33.33%) specimens showed 25≤Ct<30. Similarly, in the cultured positive samples, the bacteria growth up to the third or fourth quadrant in 33 plates, with 10(33.30%) specimens showed Ct values less than 25.

### Performances of the Respiratory Pathogens Multiplex Nucleic Acid Diagnostic Kit for bacteria detection

**Table 5** shows the concordance of the detection results for bacteria between the mPCR kit and conventional culture methods. The mPCR kit demonstrated PPA and NPA values of 84.6% (95% CI 76.6%-92.6%) and 96.5% (95% CI 96.0%-97.1%), respectively, when compared with culture. As many bacterial strains were not found by culture, positive predictive values were quite low, especially for Streptococcus pneumoniae, Haemophilus influenzae and Legionella pneumophila. By contrast, negative predictive values were very high (99.7%; 95% CI 99.5%-99.9%) for all the bacteria targets. The strongest agreement was reported with Klebsiella pneumoniae and Pseudomonas aeruginosa group.

**Table 5:** Performance summary and characteristics of the respiratory pathogens multiplex nucleic acid diagnostic kit versus those of culture used as the gold standard (the result of mPCR is positive when Ct is less than 30).

Bacterial target	No.of specimens ^a^				Performances, % (95% CI) ^b^			
	RCM+/mPCR+	RCM+/mPCR-	RCM-/mPCR+	RCM-/mPCR-	PPA	NPA	PPV	NPV
Klebsiella pneumoniae	35	4	9	683	86.2 (73.6-98.8)	97.7(96.6-98.8)	61.0(46.0-75.9)	99.4(98.8-100.0)
Streptococcus pneumoniae	7	0	45	676	100.0 (100-100)	93.2 (91.4-95.1)	5.8(-5.7-12.1)	100.0 (100-100)
Haemophilus influenzae	4	0	32	692	100.0 (100-100)	95.2 (93.6-96.7)	2.8(-2.6-8.1)	100(100-100)
Pseudomonas aeruginosa	37	4	11	676	87.9 (76.7-99.0)	97.1 (95.9-98.4)	59.2(45.4-72.9)	99.4(98.8-100)
Staphylococcus aureus	10	4	27	688	71.4 (47.8-95.1)	96.4((95.0-97.7)	27.8(13.1-42.4)	99.4(98.9-100)
Legionella pneumophila	0	0	3	725	/	/	0	100
Total	93	12	127	4140	84.6 (76.6-92.6)	96.5 (96.0-97.1)	30.6(24.2-36.7)	99.7(99.5-99.9)

a RCM, routine conventional methods; mPCR, Respiratory Pathogens Multiplex Nucleic Acid Diagnostic Kit from Sansure Biotech Inc.

b PPA, positive percent agreement; NPA, negative percent agreement; PPV, positive predictive value; NPV, negative predictive value.

## Discussion

Qualified samples are the key to the accurate detection of pathogens in lower respiratory tract infections. The detection samples of lower respiratory tract pathogens were mainly sputum, bronchial secretions and alveolar lavage fluid (Yun et al., 2019). However, there are often a large number of normally colonized pathogens in sputum and bronchial secretion, which is easy to cause specimen contamination and unqualified (Kerneis et al., 2021). Due to the deep sampling site, alveolar lavage fluid is less susceptible to colonization bacteria than sputum and bronchial secretions, the quality of samples is higher, and the detection rate of pathogens is higher than other samples (Gharsalli et al., 2018; Hogea et al., 2020). Our deliberate selection of BALF as the exclusive specimen type was driven by dual imperatives: compliance with the stringent pre-analytical requirements of multiplex pathogen detection kits (e.g., DNA/RNA stability and minimal inhibitors), and maximal exclusion of oropharyngeal commensal flora contamination—a critical advantage validated by extensive literature establishing BALF as the best specimen type for LRTI diagnosis (Chen et al., 2020). To mitigate concerns regarding bronchoscopy invasiveness, our multicenter design implemented proactive safeguards: all six participating hospitals in Hunan Province were pre-screened for high bronchoscopy throughput (>200 annual procedures), ensuring adequate residual clinical BALF samples from routine diagnostics without protocol-driven patient recruitment. This ethical sourcing approach eliminated additional procedural risks while enhancing real-world applicability.

Beyond bacterial pathogens, viral etiologies represent a significant burden in LRTIs, particularly in community-acquired pneumonia (CAP). Recent epidemiological surveillance indicates that viruses account for 22–39% of adult CAP cases globally, with influenza A/B, respiratory syncytial virus (RSV), and SARS-CoV-2 being predominant agents (Contes and Liu, 2025). The clinical significance of viral detection extends beyond primary infection: viral-bacterial coinfections occur in 15–30% of severe LRTI cases, where viruses may disrupt respiratory epithelium integrity, facilitating bacterial adhesion and exacerbating disease severity (Miyashita, 2022). Notably, influenza virus coinfection increases the mortality risk of Streptococcus pneumoniae pneumonia by 2.5-fold (Pierce et al., 2023). To further enhance the generalizability and robustness of our findings, this study incorporated a multicenter design spanning six hospitals in Hunan Province. This approach actively captures regional variations in pathogen prevalence while mitigating site-specific biases. Compared with similar studies, we found that the specimen sources of the large sample size multicenter studies were extensive, including sputum, endotracheal aspirate and bronchoalveolar lavage specimens (Gastli et al., 2021), our study uniquely combined two critical strengths: exclusive use of BALF samples to maximize detection accuracy and a multicenter framework reflecting real-world clinical diversity across institutions. However, in the study in which all samples were alveolar lavage fluid, the number of samples was only 57 (Kosai et al., 2022), our design achieved both scale (large sample size) and sample quality uniformity—significantly improving the clinical utility of pathogen identification. The multicenter data generated here provide region-specific epidemiological references for peer researchers, demonstrating how geographic and institutional heterogeneity can be systematically leveraged to strengthen external validity. By integrating rigorous sample selection with deliberate sampling diversity, this work establishes a replicable model for future studies seeking to balance methodological precision with population representativeness.

Our study demonstrated that the mPCR kit rapidly and effectively detected a variety of pathogens. The detection of bacteria was two times greater than that of the culture-based methods (**Table 2**). Previous studies have shown similar results, that is, using the same mPCR detection method to detect more bacterial targets than culture methods (Buchan et al., 2020; Lee et al., 2019). A recent study reported that mPCR method identified nearly twice as many total bacterial targets as standard-of-care culture in BAL specimens (Buchan et al., 2020). Another study reported that mPCR method detected one or more bacterial targets in an additional 20% of patients compared to culture methods (Rand et al., 2021). In addition, the overall performance of mPCR kit in our study (PPA 84.6%; NPA 96.5%) was comparable to that reported in the three small monocentric studies previously published. In the first study, 117 BAL specimens from a clinical trial (Clavel et al., 2016) were retrospectively used to evaluate the FA-PP and the authors found an overall PPA of 93.1% and NPA of 98.2% after discrepancy resolution (Yugueros-Marcos et al., 2018). In the second publication, Lee et al. studied 59 ETA and BAL specimens and reported an overall PPA of 90.0% and NPA of 97.7% with a concordance rate of 53.6% for semi-quantitative results (Lee et al., 2019). In the third study, Yoo et al. included 99 respiratory samples (sputa and ETA) and reported an overall sensitivity of 98.5% and specificity of 76.5% (Yoo et al., 2020). As in these studies, we reported a much higher proportion of samples positive for pathogens with the FA-PP than by culture (especially for Haemophilus influenzae). Indeed, the positivity rate in our study was 55.63%, similar to the previously published findings of Lee et al. (55.9%) but lower than those of Yoo et al. (72.7%) in smaller cohorts (Lee et al., 2019; Yoo et al., 2020).

The complexity of defining the sole pathogen responsible for a disease is exacerbated by the presence of co-infections. In our study, coinfections, limited to multiple bacterial pathogens, were identified in 19.8% of the specimens analyzed. However, there is a growing body of evidence regarding the incidence and pathogenesis of polymicrobial pneumonia (Babady et al., 2018; Cawcutt and Kalil, 2017; Su et al., 2019) Furthermore, recent revelations regarding the lung microbiome have challenged the traditional paradigm that the lungs are sterile and that pneumonia is exclusively caused by a single invasive pathogen (Dickson and Huffnagle, 2015). As a result, future research on pneumonia must address the complexities associated with polymicrobial respiratory disease and its implications for the pathogenesis of this condition.

In the present study, a notable discrepancy was observed between the results obtained using culture methods and the mPCR kit. Specifically, the latter method was able to detect microorganisms, including several bacterial species such as H. influenzae and S. pneumoniae, that were not detected by the culture method. This discrepancy could be attributed to several factors. Firstly, the incidence of respiratory tract infections caused by S. pneumoniae and H. influenzae may have decreased following the implementation of vaccination programs among the elderly and children. Secondly, the fastidious nature of H. influenzae, which is difficult to culture, could have contributed to the discrepancy (Sierra et al., 2020). Additionally, detection using culture methods relies on the viability of the pathogen, whereas the mPCR kit may have detected nonviable bacteria or low-abundance bacteria that were not cultured due to their fastidious growth characteristics (Buchan et al., 2020). Given these differences in characteristics between molecular assays and culture methods, it is crucial to carefully interpret the results of molecular assays when used for patient management. Future studies should further investigate the reasons for discrepancies in bacterial detection between these two methods to ensure accurate diagnosis and effective treatment of respiratory tract infections.

The mPCR kit semi-quantitatively reports typical respiratory bacterial pathogens in a timely manner, providing clinicians with crucial information about the presence and potential severity of an infection. These semi-quantitative results can be especially useful in distinguishing between colonizing bacteria and actual pathogens, as they can help guide treatment decisions. According to the instructions of the reagent kit, a positive result is indicated when the Ct value is less than 40. However, it is important to note that the results obtained using the mPCR method may differ significantly from those obtained using conventional culture methods. When the Ct value is reduced to less than 30, the mPCR detection becomes even more accurate, with a positive rate that is basically consistent with the conventional culture method. In such cases, there is a strong likelihood that the patient is infected with the target bacterium, providing clinicians with timely and actionable information. Our observation that 88.3% (265/300) of culture-negative specimens exhibited Ct values ≥30 on mPCR (**Table 4**) aligns with emerging consensus on molecular diagnostics’ interpretive challenges. Lee et al. reported similar trends in ICU patients: 68% of culture-negative BAL/ETA samples showed high Ct values (Ct>30) for bacterial targets, suggesting these likely represented low-abundance colonization or non-viable organisms rather than true infection (Lee et al., 2019). This phenomenon was further quantified by Buchan, where 74% of samples with Ct>35 correlated with bacterial loads <10^4^ CFU/mL—below typical clinical infection thresholds (Buchan et al., 2020). Crucially, our data reinforce the diagnostic gray zone concept proposed by Yugueros-Marcos: Ct values between 30-40 warrant cautious interpretation and integration with clinical parameters (Yugueros-Marcos et al., 2018). The consistency across studies highlights that: (1) Threshold standardization: Adopting pathogen-specific Ct cutoffs may improve specificity. (2) Clinical utility: Semi-quantitative Ct reporting provides actionable data for antimicrobial stewardship—high-Ct detections may justify deferred antibiotics, whereas low-Ct results demand prompt targeted therapy.

Despite its utility, there are some limitations to consider when interpreting mPCR results. Firstly, the clinical data used in this study was limited to six centers, reflecting the real-world constraints faced by clinical microbiologists in routine practice. Secondly, the interpretation of false-positive mPCR results can be challenging, as the study did not assess previous antibiotic exposure, which can significantly affect the yield of cultural methods. Additionally, some fastidious organisms may be difficult to grow under standard laboratory conditions, explaining why certain bacteria, such as Haemophilus influenzae and Streptococcus pneumoniae, were more frequently detected by the molecular approach. Critically, the technical constraints of our laboratory precluded the cultivation and isolation of Legionella pneumophila, a fastidious pathogen demanding BCYE agar (buffered charcoal yeast extract agar) with specific supplements. This limitation highlights a key vulnerability in conventional culture-based methods for intracellular pathogens. Finally, it is worth noting that the performance of mPCR for virus detection could not be evaluated in this study as standard molecular methods were not performed for all positive specimens.

## Data Availability

The raw data supporting the conclusions of this article will be made available by the authors, without undue reservation.

## References

[B1] Babady N. E. England M. R. Jurcic Smith K. L. He T. Wijetunge D. S. Tang Y. W.. (2018). Multicenter evaluation of the ePlex respiratory pathogen panel for the detection of viral and bacterial respiratory tract pathogens in nasopharyngeal swabs. J. Clin. Microbiol. 56. doi: 10.1128/JCM.01658-17, PMID: 29212701 PMC5786739

[B2] Buchan B. W. Windham S. Balada-Llasat J. M. Leber A. Harrington A. Relich R.. (2020). Practical comparison of the bioFire filmArray pneumonia panel to routine diagnostic methods and potential impact on antimicrobial stewardship in adult hospitalized patients with lower respiratory tract infections. J. Clin. Microbiol. 24, 58. doi: 10.1128/JCM.00135-20, PMID: 32350045 PMC7315039

[B3] Cawcutt K. Kalil A. C. (2017). Pneumonia with bacterial and viral coinfection. Curr. Opin. Crit. Care 23, 385–390. doi: 10.1097/MCC.0000000000000435, PMID: 28777158

[B4] Chen H. Yin Y. Gao H. Guo Y. Dong Z. Wang X.. (2020). Clinical utility of in-house metagenomic next-generation sequencing for the diagnosis of lower respiratory tract infections and analysis of the host immune response. Clin. Infect. Dis. 71, S416–S426. doi: 10.1093/cid/ciaa1516, PMID: 33367583

[B5] Clavel M. Barraud O. Moucadel V. Meynier F. Karam E. Ploy M. C.. (2016). Molecular quantification of bacteria from respiratory samples in patients with suspected ventilator-associated pneumonia. Clin. Microbiol. Infect. 22, 812.e811–812 e817. doi: 10.1016/j.cmi.2016.06.013, PMID: 27404367

[B6] Collaborators GBDLRI. (2018). Estimates of the global, regional, and national morbidity, mortality, and aetiologies of lower respiratory infections in 195 countrie-2016: a systematic analysis for the Global Burden of Disease Study 2016. Lancet Infect. Dis. 18, 1191–1210. doi: 10.1016/S1473-3099(18)30310-4, PMID: 30243584 PMC6202443

[B7] Contes K. M. Liu B. M. (2025). Epidemiology, clinical significance, and diagnosis of respiratory viruses and their co-infections in the post-COVID era. Pathogens. Mar. 7, 14. doi: 10.3390/pathogens14030262, PMID: 40137747 PMC11944763

[B8] Dickson R. P. Huffnagle G. B. (2015). The lung microbiome: new principles for respiratory bacteriology in health and disease. PloS Pathog. 11, e1004923. doi: 10.1371/journal.ppat.1004923, PMID: 26158874 PMC4497592

[B9] Gastli N. Loubinoux J. Daragon M. Lavigne J. P. Saint-Sardos P. Pailhories H.. (2021). Multicentric evaluation of BioFire FilmArray Pneumonia Panel for rapid bacteriological documentation of pneumonia. Clin. Microbiol. Infect. 27, 1308–1314. doi: 10.1016/j.cmi.2020.11.014, PMID: 33276137

[B10] Gharsalli H. Mlika M. Sahnoun I. Maalej S. Douik El Gharbi L. Mezni F. E. (2018). The utility of bronchoalveolar lavage in the evaluation of interstitial lung diseases: A clinicopathological perspective. Semin. Diagn. Pathol. 35, 280–287. doi: 10.1053/j.semdp.2018.08.003, PMID: 30173880

[B11] Hogea S. P. Tudorache E. Pescaru C. Marc M. Oancea C. (2020). Bronchoalveolar lavage: role in the evaluation of pulmonary interstitial disease. Expert Rev. Respir. Med. 14, 1117–1130. doi: 10.1080/17476348.2020.1806063, PMID: 32847429

[B12] Iregui M. Ward S. Sherman G. Fraser V. J. Kollef M. H. (2002). Clinical importance of delays in the initiation of appropriate antibiotic treatment for ventilator-associated pneumonia. Chest 122, 262–268. doi: 10.1378/chest.122.1.262, PMID: 12114368

[B13] Kerneis S. Visseaux B. Armand-Lefevre L. Timsit J. F. (2021). Molecular diagnostic methods for pneumonia: how can they be applied in practice? Curr. Opin. Infect. Dis. 34, 118–125. doi: 10.1097/QCO.0000000000000713, PMID: 33395094

[B14] Kosai K. Akamatsu N. Ota K. Mitsumoto-Kaseida F. Sakamoto K. Hasegawa H.. (2022). BioFire FilmArray Pneumonia Panel enhances detection of pathogens and antimicrobial resistance in lower respiratory tract specimens. Ann. Clin. Microbiol. Antimicrob. 21, 24. doi: 10.1186/s12941-022-00512-8, PMID: 35659683 PMC9166201

[B15] Lee S. H. Ruan S. Y. Pan S. C. Lee T. F. Chien J. Y. Hsueh P. R. (2019). Performance of a multiplex PCR pneumonia panel for the identification of respiratory pathogens and the main determinants of resistance from the lower respiratory tract specimens of adult patients in intensive care units. J. Microbiol. Immunol. Infect. 52, 920–928. doi: 10.1016/j.jmii.2019.10.009, PMID: 31806539 PMC7185395

[B16] Miyashita N. (2022). Atypical pneumonia: Pathophysiology, diagnosis, and treatment. Respir. Investig. 60, 56–67. doi: 10.1016/j.resinv.2021.09.009, PMID: 34750083

[B17] Pierce V. M. Bhowmick T. Simner P. J. (2023). Guiding antimicrobial stewardship through thoughtful antimicrobial susceptibility testing and reporting strategies: an updated approach in 2023. J. Clin. Microbiol. 61, e0007422. doi: 10.1128/jcm.00074-22, PMID: 37768094 PMC10662363

[B18] Prats E. Dorca J. Pujol M. Garcia L. Barreiro B. Verdaguer R.. (2002). Effects of antibiotics on protected specimen brush sampling in ventilator-associated pneumonia. Eur. Respir. J. 19, 944–951. doi: 10.1183/09031936.02.00239302, PMID: 12030737

[B19] Ramanan P. Bryson A. L. Binnicker M. J. Pritt B. S. Patel R. (2018). Syndromic panel-based testing in clinical microbiology. Clin. Microbiol. Rev. 31. doi: 10.1128/CMR.00024-17, PMID: 29142077 PMC5740973

[B20] Rand K. H. Beal S. G. Cherabuddi K. Couturier B. Lingenfelter B. Rindlisbacher C.. (2021). Performance of a semiquantitative multiplex bacterial and viral PCR panel compared with standard microbiological laboratory results: 396 patients studied with the bioFire pneumonia panel. Open Forum Infect. Dis. 8, ofaa560. doi: 10.1093/ofid/ofaa560, PMID: 33447631 PMC7793460

[B21] Sierra Y. Tubau F. Gonzalez-Diaz A. Carrera-Salinas A. Moleres J. Bajanca-Lavado P.. (2020). Assessment of trimethoprim-sulfamethoxazole susceptibility testing methods for fastidious Haemophilus spp. Clin. Microbiol. Infect. 26, 944 e941–944 e947. doi: 10.1016/j.cmi.2019.11.022, PMID: 31811916

[B22] Su I. C. Lee K. L. Liu H. Y. Chuang H. C. Chen L. Y. Lee Y. J. (2019). Severe community-acquired pneumonia due to Pseudomonas aeruginosa coinfection in an influenza A(H1N1)pdm09 patient. J. Microbiol. Immunol. Infect. 52, 365–366. doi: 10.1016/j.jmii.2018.05.007, PMID: 29958866

[B23] Torres A. Lee N. Cilloniz C. Vila J. van der Eerden M. (2016). Laboratory diagnosis of pneumonia in the molecular age. Eur. Respir. J. 48, 1764–1778. doi: 10.1183/13993003.01144-2016, PMID: 27811073

[B24] Yoo I. Y. Huh K. Shim H. J. Yun S. A. Chung Y. N. Kang O. K.. (2020). Evaluation of the BioFire FilmArray Pneumonia Panel for rapid detection of respiratory bacterial pathogens and antibiotic resistance genes in sputum and endotracheal aspirate specimens. I. nt J. Infect. Dis. 95, 326–331. doi: 10.1016/j.ijid.2020.03.024, PMID: 32179139

[B25] Yugueros-Marcos J. Barraud O. Iannello A. Ploy M. C. Ginocchio C. Rogatcheva M.. (2018). New molecular semi-quantification tool provides reliable microbiological evidence for pulmonary infection. Intensive Care Med. 44, 2302–2304. doi: 10.1007/s00134-018-5417-0, PMID: 30350171

[B26] Yun K. W. Wallihan R. Juergensen A. Mejias A. Ramilo O. (2019). Community-acquired pneumonia in children: myths and facts. Am. J. Perinatol. 36, S54–S57. doi: 10.1055/s-0039-1691801, PMID: 31238360

