# Supplementary material for: Multicenter evaluation of fast multiplex PCR for detection of pathogens in lower respiratory tract infections
Source: Front Cell Infect Microbiol. 2025 Oct 2;15:1643991. doi: 10.3389/fcimb.2025.1643991 (PMC12528164; doi:10.3389/fcimb.2025.1643991)
Supplement: Supplementary file 1 [file Table1.docx]

**Table S1**

Bacteria identified using the Respiratory Pathogens Multiplex Nucleic Acid Diagnostic Kit (Ct<40) and culture-based methods.

| **Category** | **RCM+, n (%)** | **PCR+(Ct<40), n (%)** |
| --- | --- | --- |
| **Bacterium** |  |  |
| Pseudomonas aeruginosa | 41(39.0%) | 96(15.3%) |
| Klebsiella pneumoniae | 39(37.1%) | 110(17.5%) |
| Staphylococcus aureus | 14(13.3%) | 37(5.9%) |
| Streptococcus pneumoniae | 7(6.7%) | 219(34.9%) |
| Haemophilus influenzae | 4(3.8%) | 94(15.0%) |
| Legionella pneumophila | 0 | 3(0.5%) |
| **Total** | 105 | 559 |
