# Supplementary material for: Multicenter evaluation of fast multiplex PCR for detection of pathogens in lower respiratory tract infections
Source: Front Cell Infect Microbiol. 2025 Oct 2;15:1643991. doi: 10.3389/fcimb.2025.1643991 (PMC12528164; doi:10.3389/fcimb.2025.1643991)
Supplement: Supplementary file 2 [file Table2.docx]

**Table S2**

| **Sample Number** | **Pathogens** |
| --- | --- |
| 22 | Haemophilus influenzae |
| 30 | Haemophilus influenzae |
| 619 | Haemophilus influenza |
| 261 | Streptococcus pneumoniae |
| 382 | Streptococcus pneumonia + Haemophilus influenza |
| 617 | Streptococcus pneumoniae |
| 685 | Streptococcus pneumoniae |
| 712 | Streptococcus pneumoniae |
| 727 | Streptococcus pneumoniae |
| 728 | Streptococcus pneumoniae |
| 46 | Legionella pneumophila+ Pseudomonas aeruginosa |
| 61 | Legionella pneumophila+Influenza A virus |
| 307 | Legionella pneumophila |

Co testing mode of harsh cultivation bacteria
